# Manufacturing of stretchable substrate with biaxial strain control for highly-efficient stretchable solar cells and displays

**DOI:** 10.1038/s41598-023-47569-9

**Published:** 2023-11-22

**Authors:** Ji-Youn Kwak, Ji-Young Jeong, Ye-Pil Kwon, Dong-Hyun Seo, Chung-Mo Kang, Dong-Hyeon Kim, Jun Sae Han, Eun-Ji Gwak, Doo-Sun Choi, Ju-Young Kim, Tae-Jin Je, Eun-chae Jeon

**Affiliations:** 1 School of Materials Science and Engineering, University of Ulsan, Ulsan, 44610 Republic of Korea; 2 Department of Materials Science and Engineering, Ulsan National Institute of Science and Technology (UNIST), Ulsan, 44919 Republic of Korea; 3 Department of Nano Manufacturing Technology, Korea Institute of Machinery & Materials (KIMM), Daejeon, 34141 Republic of Korea; 4 Department of Nano-Mechatronics Engineering, University of Science & Technology (UST), Daejeon, 34113 Republic of Korea

**Keywords:** Mechanical properties, Mechanical engineering

## Abstract

There has been significant research focused on the development of stretchable materials that can provide a large area with minimal material usage for use in solar cells and displays. However, most materials exhibit perpendicular shrinkage when stretched, which is particularly problematic for polymer-based substrates commonly used in stretchable devices. To address this issue, biaxial strain-controlled substrates have been proposed as a solution to increase device efficiency and conserve material resources. In this study, we present the design and fabrication of a biaxial strain-controlled substrate with a re-entrant honeycomb structure and a negative Poisson’s ratio. Using a precisely machined mold with a shape error of less than 0.15%, we successfully fabricated polydimethylsiloxane substrates with a 500 μm thick re-entrant honeycomb structure, resulting in a 19.1% reduction in perpendicular shrinkage. This improvement translates to a potential increase in device efficiency by 9.44% and an 8.60% reduction in material usage for substrate fabrication. We demonstrate that this design and manufacturing method can be applied to the fabrication of efficient stretchable devices, such as solar cells and displays.

## Introduction

Flexible devices, capable of changing shape as needed, have gained significant attention across various industrial fields^1–3^. Although flexible devices are available in various form factors, their area remains constant even when bent, curved, folded, or rolled. In contrast, stretchable devices have the advantage of variable area, and users typically increase their area during use^4–6^. Notably, if a solar cell, whose performance is directly related to its area, is stretchable, it can generate more electricity under given sunlight conditions and with a given amount of materials. Therefore, stretchability can enhance device efficiency and save resources. This has practical applications in the development of portable solar cells for mobile devices. Moreover, this technology is expected to find utility in displays including portable camping lights. Additionally, stretchable devices have gained prominence in sensor technology, serving as fundamental components across a wide array of applications, including deformable sensors^7–9^ and standalone sensing platforms^10^. The key performance parameter of stretchable (solar cell) devices is the extent to which the area can be increased by users during operation, known as “stretchability.” In previous studies^3,6,11,12^, the strain in the stretching direction (generally longitudinal direction) has been used as an indicator of the stretchability. However, most natural materials shrink in the transverse direction when stretched longitudinally. Therefore, although the longitudinal strain may increase by 100%, the increase in the area is less than 100%, requiring a larger amount of materials to achieve a 100% increase in the area. In particular, polymer materials commonly used for fabricating substrates in stretchable solar cells exhibit high perpendicular shrinkage, which reduces efficiency. Hence, biaxial strain control (longitudinal and transverse strain) of the substrate is necessary to achieve high efficiency and conserve resources in these devices.

The control of transverse strain during the longitudinal stretching of a material is known as biaxial strain-control. One approach to achieve such control is by utilizing a material possessing an auxetic structure, such as re-entrant type^13,14^, arrow type^15,16^, chiral type^17,18^, and kirigami/origami^19,20^. An auxetic structure can minimize perpendicular shrinkage while allowing for perpendicular expansion through appropriate material design, without altering the inherent properties^14,15^. Thus, the use of an auxetic structure enables the production of large areas of material with a reduced amount of resources. The other approach to achieve biaxial strain-control is surface relief structures, such as tripod^21^, trapezoidal^22^, and square prism^23^. Surface relief structures are wavy banded structures made from plates with specific patterns or geometrics that, when stretched, straighten out where they were bent to achieve biaxial strain-control. However, the complex shapes of auxetic structures and other surface relief structures necessitate their fabrication via time-consuming and costly methods such as additive manufacturing^24–27^, stereolithography^28–30^, laser ablation^31,32^, or chemical etching^33^. Several studies have explored cost-effective and time-efficient fabrication methods for auxetic structures, such as fabricating and assembling different non-auxetic structures^34^, folding plates into auxetic shapes^35^, and so on^30^. In this study, we present a method for manufacturing biaxial strain-controlled substrates with an auxetic structure, which can be utilized for stretchable solar cells and displays. We produced a negative-patterned auxetic structure in a metal mold using precise micro end-milling and subsequently utilized a replication process to fabricate biaxial strain-controlled stretchable substrates. With this technique, substrates with a large surface area could be produced utilizing a limited amount of materials.

## Design and manufacturing of biaxial strain-controlled stretchable substrates

**Figure 1:**
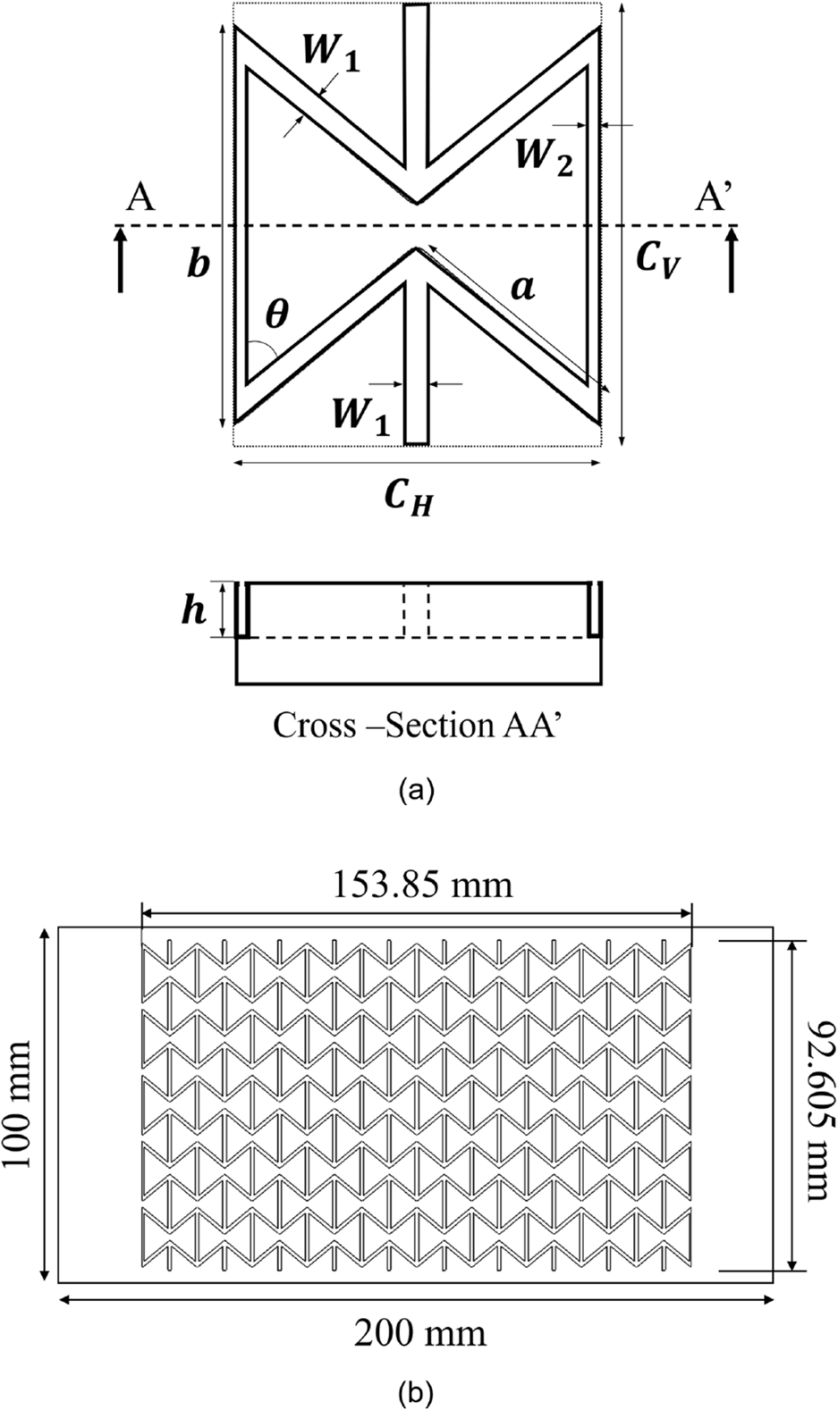
A schematic diagram of (**a**) the unit cell of the re-entrant honeycomb structure type I and a cross-sectional image of the machined metal mold and (**b**) machining of negative patterned re-entrant honeycomb structure on a brass metal mold using precise micro end-milling.

Biaxial strain-controlled characteristics were evaluated on the basis of Poisson’s ratio, which is one of the inherent properties of materials. As shown in Eq. (1), Poisson’s ratio (ν) is the ratio of the longitudinal strain (ε_l_) to the transverse strain (ε_t_). Defining stretchability as the ratio of area increase, it can be expressed as the ratio of the area before stretching (A_0_) to the area after stretching (A_1_), as shown in Eq. (2). Therefore, the smaller Poisson’s ratio, the better stretchability (corresponding to the biaxial strain-controlled characteristics). We considered three auxetic structures—type I and type II re-entrant honeycomb structures and a double arrow structure—that could have the widest range of Poisson’s ratio and that had a relatively simple two-dimensional shape, which made it easy to machine them in the mold. On the basis of previous studies^36–38^, unit cell design codes for the three auxetic structures were generated on Matlab software, with the Poisson’s ratio (ν) and maximum strain (ε_l.max_) as the variables. As the Poisson’s ratio is inversely proportional to biaxial strain-controlled characteristics and as the maximum strain is a critical parameter of stretchable solar cell substrates, we designed structures with various Poisson’s ratios and maximum strain values of 30% or more by using the generated design codes. Additionally, in consideration of the size and shape of the unit cell and need to engrave the mold, the structure chosen for being investigated in detail was a re-entrant honeycomb structure of type I with a Poisson’s ratio of -1 and a maximum strain of 30%. The unit cell of the structure is shown in Fig. 1a, and its parameters are presented in Table 1. It had biaxial strain-controlled characteristics and the lateral parts extended outward when the structure was stretched. When designing a unit cell of a re-entrant honeycomb structure of type I, increasing μ (C_H_/C_V_) or decreasing θ can result in improved biaxial strain-controlled characteristics. However, excessively large μ values could lead to diagonal lines coalescence, and excessive θ reduction might cause diagonal and horizontal lines merging. Therefore, it’s essential to determine an appropriate value for these parameters. Thus, determining appropriate parameter values is critical. To address these factors and others, we developed a MATLAB code to calculate optimal values, guiding the unit cell design presented in Table 1. Finally, an auxetic structure comprising fifty-unit cells was designed for machining in a brass mold with a length of 200 mm and a width of 100 mm, as shown in Fig. 1b.

**Table 1:** Details of the re-entrant honeycomb unit cell with a Poisson’s ratio of − 1 and a maximum strain of 30%.

Parameters	Designed values
C_H_	15.385 mm
C_V_	18.521 mm
μ (C_H_/C_V_)	0.83068
a	10 mm
b	15.650 mm
θ	50.285°
W_1_ (= 2W_2_)	1 mm
h	0.5 mm

1 $$\nu =-\frac{{\varepsilon }_{t}}{{\varepsilon }_{l}},$$

2 $$\frac{{A}_{1}}{{A}_{0}}=\frac{{A}_{0}(1+{\varepsilon }_{l})(1+{\varepsilon }_{t})}{{A}_{0}}=\left(1+{\varepsilon }_{l}\right)\left(1+{\varepsilon }_{t}\right)=\left(1+{\varepsilon }_{l}\right)\left(1-\nu {\varepsilon }_{l}\right).$$

**Figure 2:**
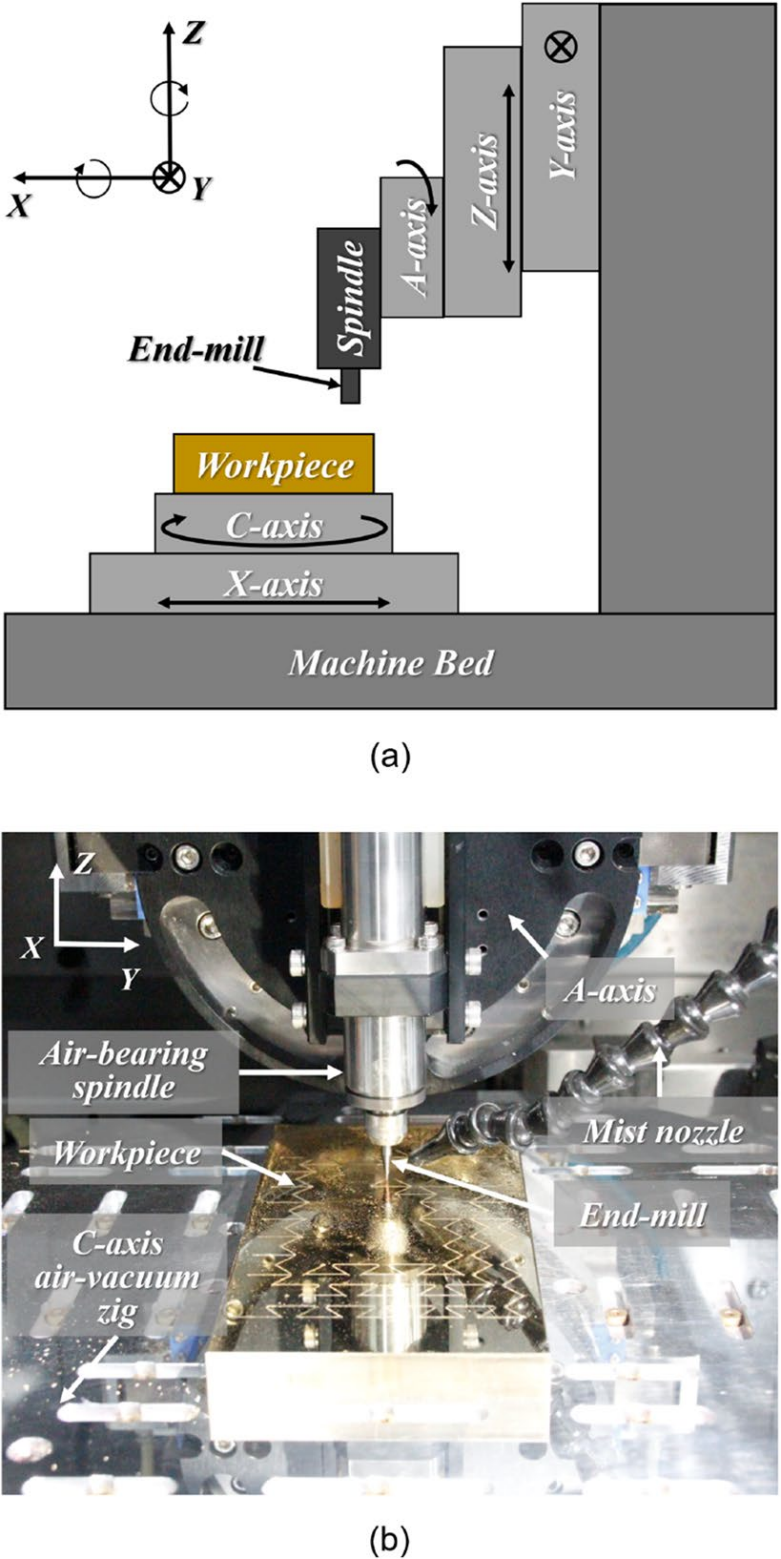
(**a**) A schematic of ultra-precision 5-axis end-milling system and (**b**) a set-up of ultra-precision end-milling system for fabricating a metal mold with a negative-patterned re-entrant honeycomb structure.

Figure 2 shows a schematic and a setup image of the machine tools (UVM-450, TOSHIBA) used for fabricating a metal mold with a negative-patterned re-entrant honeycomb structure. The machine tools consisted of three linear stages (*x*-, *y*-, and *z*-axis) with a resolution of 10 nm, two rotary stages (C- and A-axis) with a resolution of 0.00001°, and an electric motor spindle (E3000, NAKANISHI) that had a maximum rotational speed of 60,000 rpm. In order to fabricate a structure that is advantageous for releasing a stretchable substrate mold from a machined metal mold, an end-mill tool (MTE230, NS TOOL) with a diameter of 500 μm and a taper angle of 10° was used. The machining conditions used in this study are presented in Table 2. The rotational speed of the electronic spindle was 50,000 rpm, the feed rate of the end-mill was which could be 200 mm/min which could be calculated by 5 μm/tooth, and the pitch was about 163.3 μm. The workpiece material used was 64 brass, and it was pre-machined to a flatness of 0.2 μm and a surface roughness of about 20 nm for obtaining a uniform machining depth in the work area.

**Table 2:** Details of machining conditions of the re-entrant honeycomb structure on a brass mold.

Machining parameter		Value
End-mill spindle rotational speed		50,000 rev/min
Feed rate		200 mm/min
Cutting pitch	Rough	163.3 μm/pass
	Finish	10 μm/pass
Depth of cut	First and finish	5 μm/pass
	Rough	98 μm/passes × 5 passes

**Figure 3:**
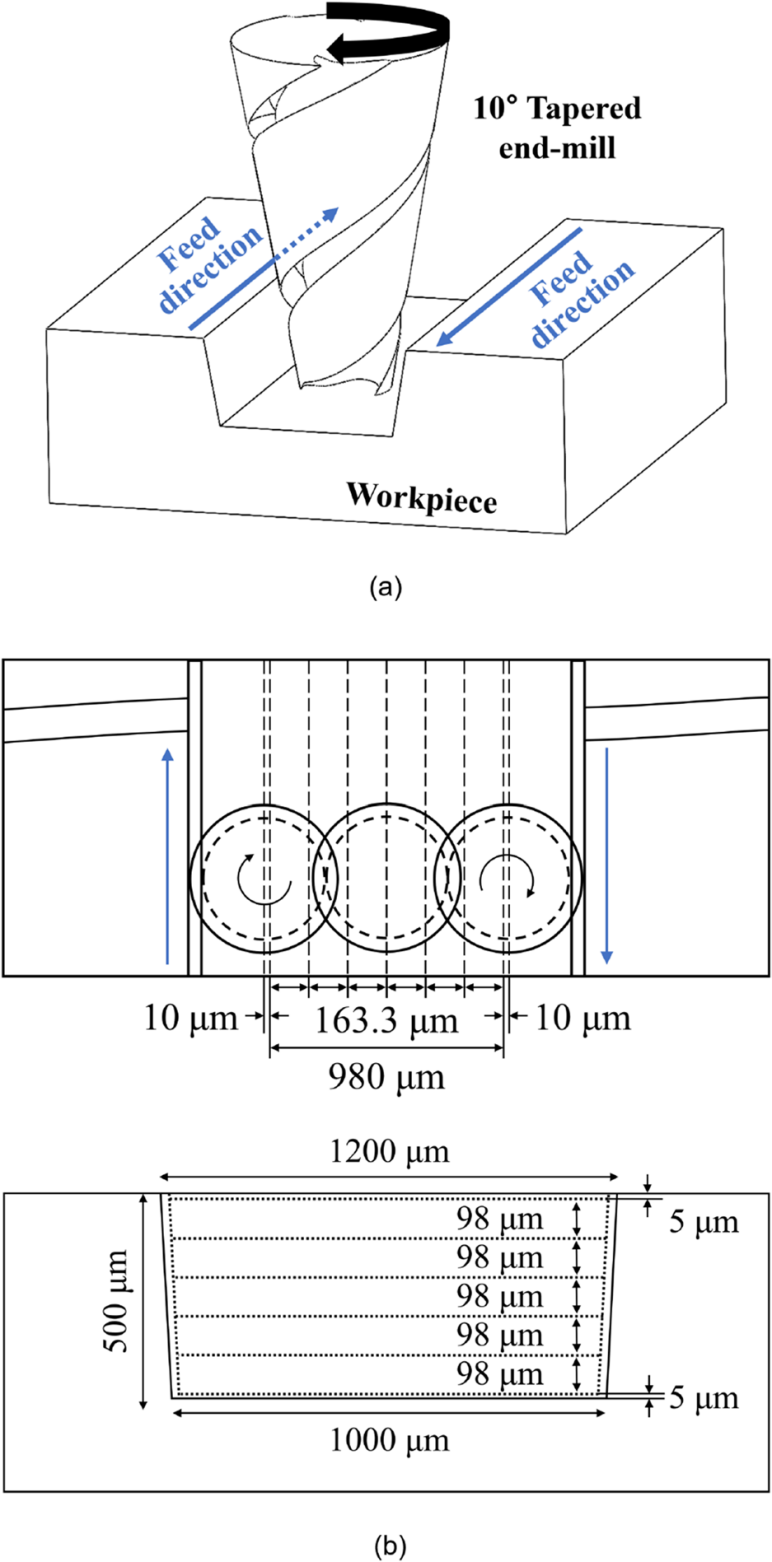
(**a**) Geometric of the cutting process using tapered end-mill and (**b**) a schematic of the end-milling process for machining the metal mold with a negative patterned re-entrant honeycomb structure.

Figure 3 shows the machining method used for the re-entrant honeycomb structure on a metal mold. The cutting tool path of the designed re-entrant honeycomb structure was generated using SOLIDWORKS CAM software. All machining processes involved down-milling according to the designed cutting tool path for minimizing the roughness and burr size^39–41^. Furthermore, the machining process was divided into rough cutting and outline cutting. In the rough cutting process, relatively rough machining conditions with a cutting pitch of 163.3 μm/pass and a cutting depth of 98 μm/pass were used to rapidly remove material and reduce machining time. Outline cutting was then performed with a width of 10 μm/pass from a designed sidewall to improve the quality of the machined surface. The cutting depth was set to a small value, namely 5 μm/pass, in the initial and final cutting in order to minimize burrs, which easily occur at the edges of the machined pattern, and improve the surface quality.

After machining the metal mold with a negative-patterned re-entrant honeycomb structure, we fabricated a biaxial strain-controlled stretchable substrate by using polydimethylsiloxane (PDMS) and a replication process. This manufacturing approach using a metal mold and a replication process for stretchable substrates is applicable to any soft materials, provided there is a non-adhesive curing method against a metal mold or the utilization of an additional release agent. Among various soft materials, PDMS is widely used for fabricating substrates of stretchable electronic devices because of its advantages, which include stable chemical properties, good thermal stability, high transparency, and high biological compatibility^5, 42^. PDMS (Sylgard 184, Dow Corning) was prepared by mixing a base and a curing agent in a 10:1 weight ratio. The mixture was poured into the machined metal mold with a negative-patterned re-entrant honeycomb structure and cured at 100 °C for 25 min. The cured PDMS was removed from the mold, and it was used as a molded stretchable substrate with a positive-patterned re-entrant honeycomb structure. In the fabrication of stretchable substrates with an auxetic structure through the replication process, the fabrication cost can be reduced by reusing the machined metal mold, and the time is considerably shorter compared with conventional methods such as additive manufacturing and stereolithography.

## Results and discussion

### A machined metal mold and molded biaxial strain-controlled stretchable substrates

**Figure 4:**
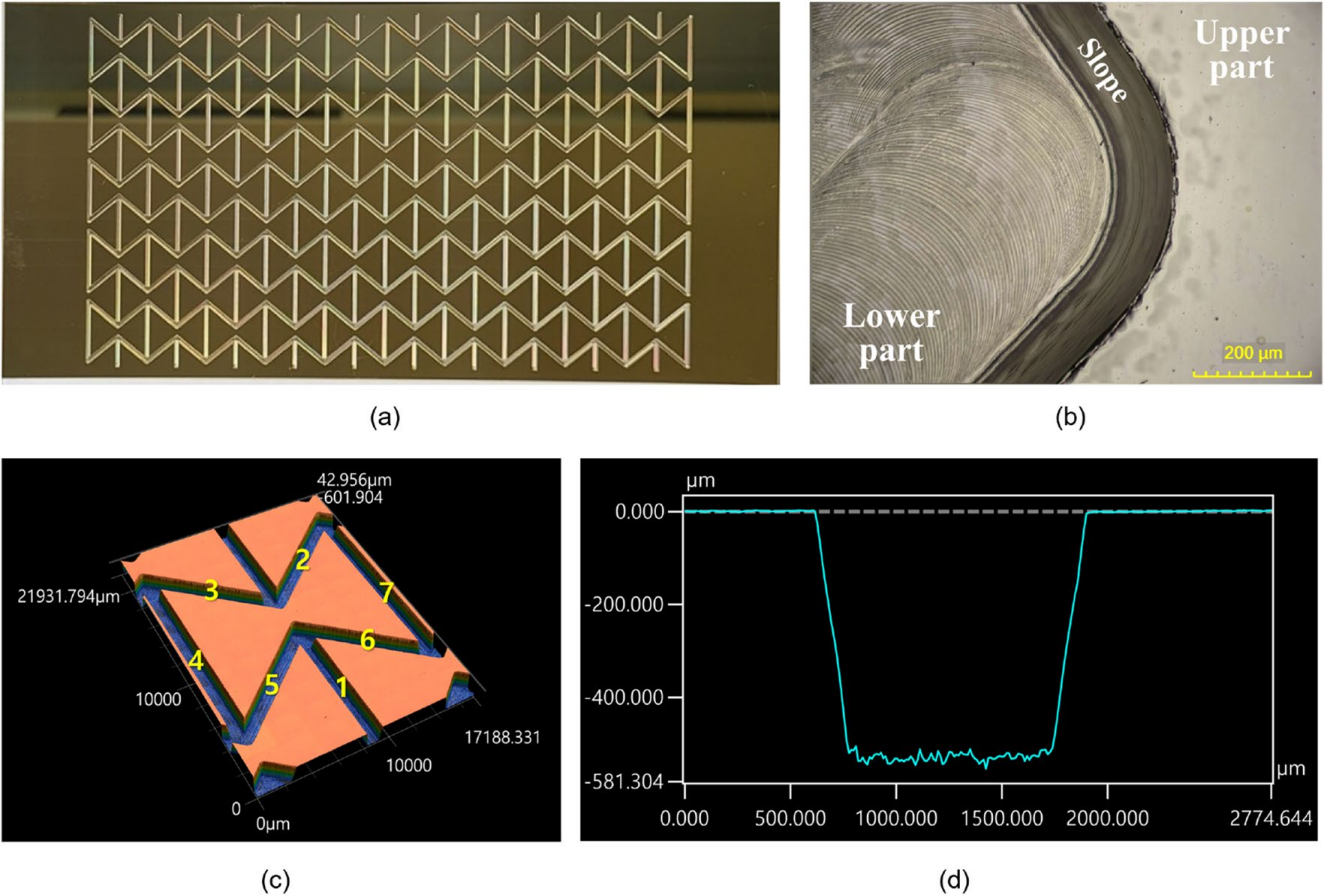
(**a**) The machined metal mold with a negative patterned re-entrant honeycomb structure, (**b**) a surface of the machined metal mold observed by optical microscope and (**c**) the machined re-entrant honeycomb structure observed by a confocal laser scanning microscope and the results of profile measurement of (**d**) line 2.

As shown in Fig. 4a, a brass mold machined through precise micro end-milling comprised fifty re-entrant honeycomb unit cells, as designed in Fig. 1b. The surface of the mold was observed through an optical microscope, and it is shown in Fig. 4b. Only the lower part of the brass mold was machined, and this part had a negative-patterned re-entrant honeycomb structure in the brass mold; the upper part was not machined. Furthermore, there are slopes between the lower and upper parts since the tool used had a taper angle of 10°. If abnormal deformations, fractures, and/or large burrs, which could interrupt the replication process, had occurred during the precise micro end-milling processes, they would have been easily spotted. However, there were no defects over the entire mold of the machined surfaces, as evident in Fig. 4a and b. To determine whether the metal mold with a re-entrant honeycomb structure had been precisely machined to have the designed dimensions, we observed the structure using a confocal laser scanning microscope. Seven different locations within a single unit cell, shown in Fig. 4c, were analyzed, and one of them is shown in Fig. 4d. The average of the lower and upper widths of the patterns in the brass mold was 1002.10 μm and 1213.14 μm, respectively, and the shape error was less than 0.15%. Thus, the metal mold with a negative-patterned re-entrant honeycomb structure had been well machined.

**Figure 5:**
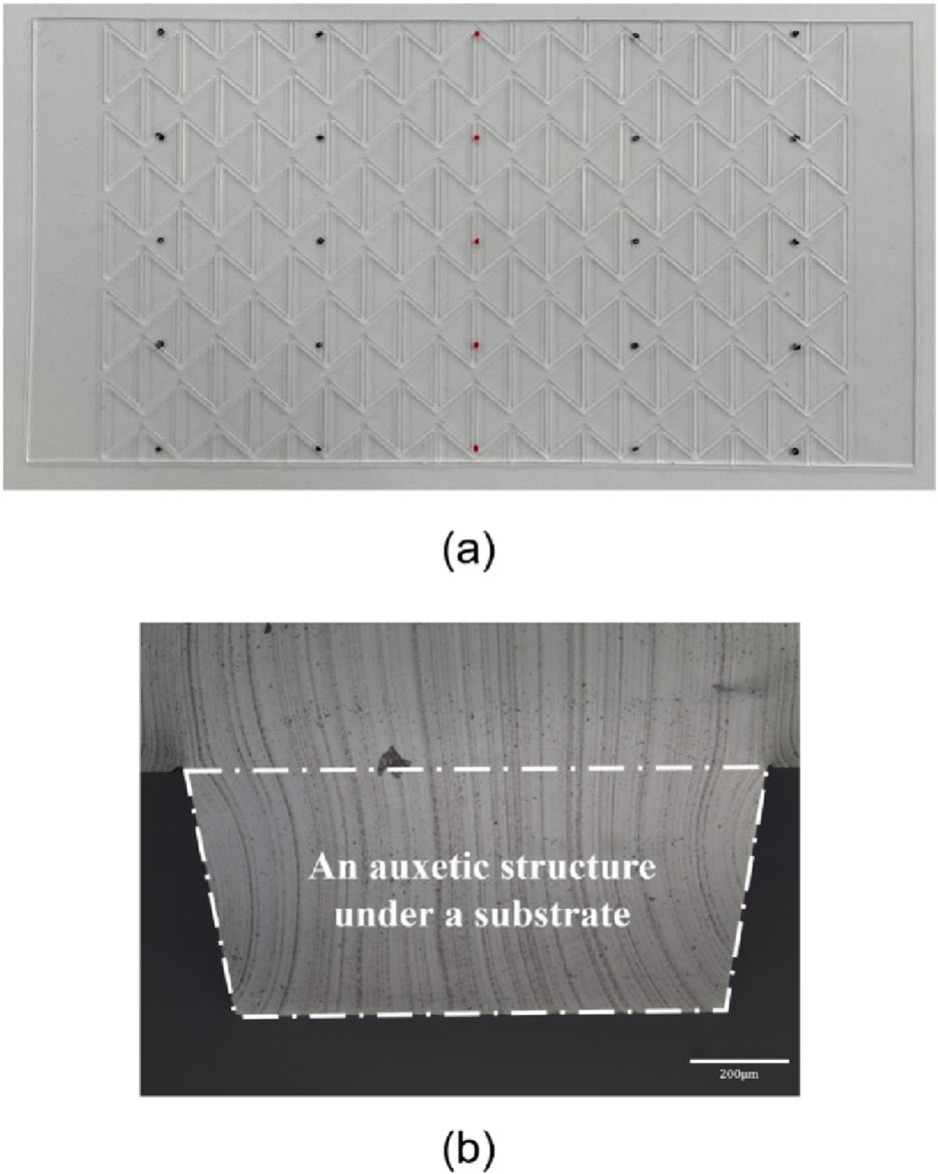
(**a**) The molded PDMS substrate with 500μm-thick re-entrant honeycomb structure and (**b**) a cross-sectional image of a positive re-entrant honeycomb structure in a molded PDMS substrate observed by an optical microscope.

The molded PDMS stretchable substrates were fabricated well by using the machined metal mold with a negative-patterned re-entrant honeycomb structure and a replication process without any tearing, as shown in Fig. 5a. To verify whether the molded auxetic structure conformed to the designed dimensions of the mold, we observed a cross-sectional image of a positive re-entrant honeycomb structure in a molded PDMS stretchable substrate by using an optical microscope; the cross-sectional image is shown in Fig. 5b. The lower width, upper width, and height of the positive re-entrant honeycomb structure were about 957.88, 1164.52, and 491.65 μm, respectively, and they were smaller than the designed values of the machined metal mold by 4.41%, 4.01%, and 5.45%, respectively. This difference is related to the shrinkage of PDMS during the curing process. When PDMS is cured at a high temperature, a monomer of PDMS is cross-linked and the total volume is reduced^43^. Since the height had little effect on the Poisson’s ratio in the design code developed in this study and since the differences in the lower and upper width were less than 5%, the molded stretchable substrates had shapes very similar to the designed shapes.

### Characterization of biaxial strain-controlled stretchable substrates

As mentioned, theoretically, an auxetic structure has biaxial strain-controlled characteristics when it is not combined with any material. However, the molded stretchable substrate fabricated using a metal mold and a replication process had an auxetic structure combined with a planar substrate. Hence, it could be expected to have a Poisson’s ratio value between the negative Poisson’s ratio of the auxetic structure and the positive Poisson’s ratio of the planar substrate. Thus, it was necessary to evaluate whether the molded stretchable substrate fabricated by the method we developed had biaxial strain-controlled characteristics. Toward this end, we compared the Poisson’s ratio of a planar substrate without an auxetic structure and one with an auxetic structure. Furthermore, to investigate the effect of the thickness of the planar substrate combined with the auxetic structure, planar substrates with thickness of 1.0, 1.2, 1.5, and 1.8 mm were fabricated and combined (one at a time) with the 500 μm thick auxetic structure.

**Figure 6:**
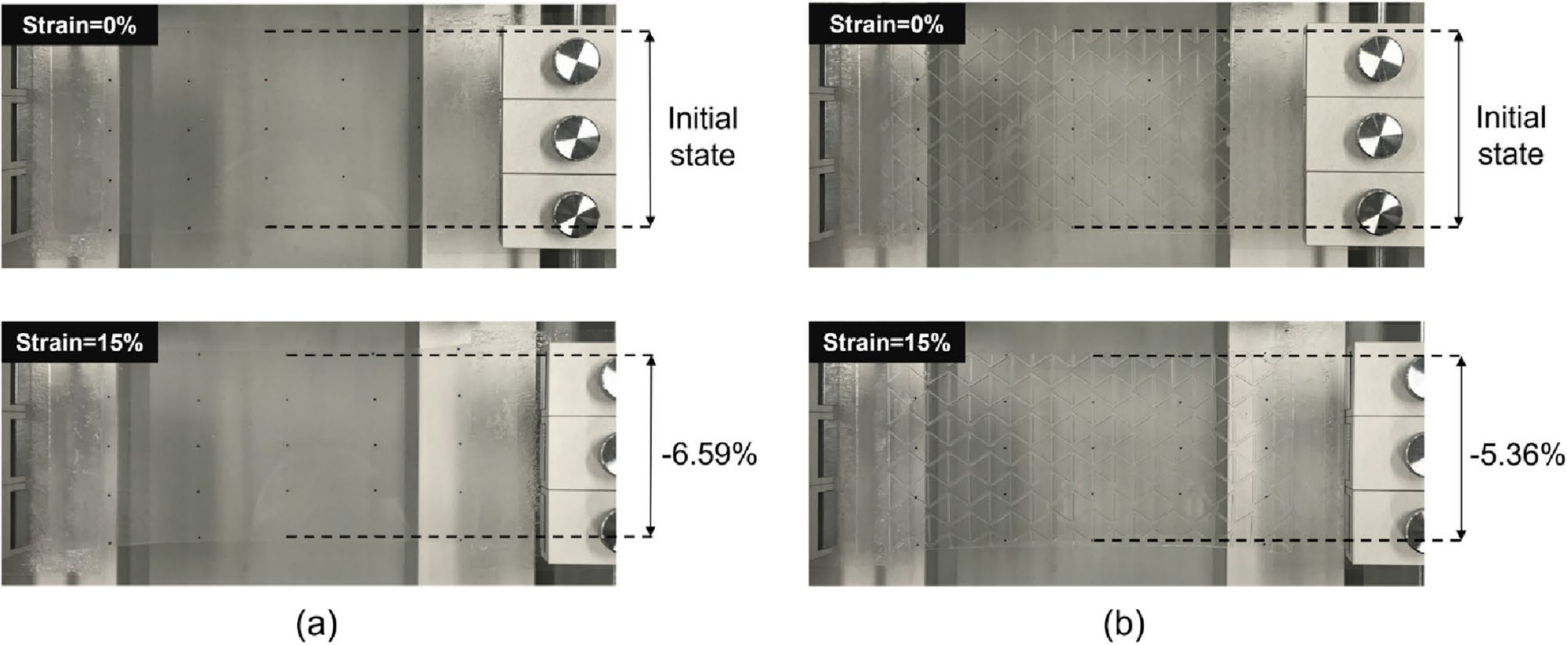
(**a**) The 1.2mm-thick planar PDMS substrate without an auxetic structure and (**b**) the 1.2mm-thick planar PDMS substrate with a 500μm-thick re-entrant honeycomb structure.

**Figure 7:**
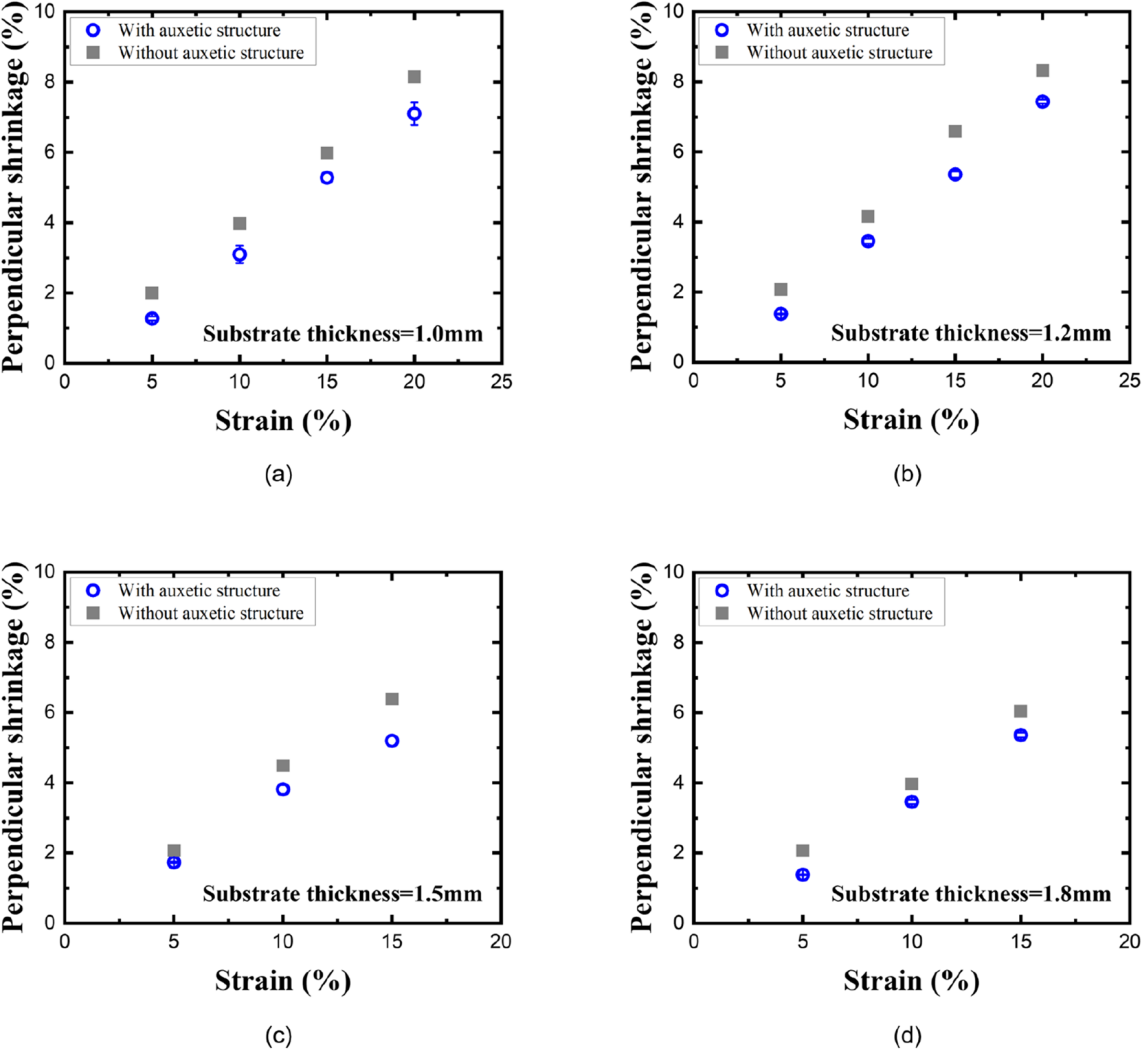
The variation of perpendicular shrinkage with the longitudinal strain when the thickness of the planar substrate is (**a**) 1.0 mm, (**b**) 1.2 mm, (**c**) 1.5 mm, and (**d**) 1.8 mm.

Since the Poisson’s ratio is obtained from the longitudinal strain and transverse strain as shown in Eq. (1), it can be compared by the perpendicular shrinkage for the same longitudinal strain based on the lattice points on the specimen, as shown in Fig. 5a. Specimens with 1.0, 1.2, 1.5, and 1.8 mm thick planar substrates were stretched by a longitudinal strain of 15%–20%. We compared the width ratio of a 1.2 mm thick planar PDMS substrate without an auxetic structure and a 1.2 mm thick planar PDMS substrate with a 500 μm thick re-entrant honeycomb structure (Fig. 6). The width ratio of the 1.2 mm thick planar PDMS substrate without an auxetic structure was reduced by 6.59% at a 15% longitudinal strain. Under the same longitudinal strain, the width ratio of the 1.2 mm thick planar PDMS substrate with a 500 μm thick re-entrant honeycomb structure was reduced by 5.36%. The perpendicular shrinkage decreased by about 18.7% when the 500 μm thick re-entrant honeycomb structure was introduced, and a decrease in the perpendicular shrinkage was also observed at different strains and thicknesses. Figure 7 shows the variation of the perpendicular shrinkage with the longitudinal strain for different thickness of the planar substrate, and the slope of graphs gives the Poisson’s ratio. The Poisson’s ratio decreased when the 500 μm thick re-entrant honeycomb structure was introduced, regardless of the thickness of the planar substrate. The Poisson’s ratio of a material should have a constant value as it is an inherent property of the material. Hence, if an appropriate assessment had been made, all graphs should have been a linear function passing through the zero point. To verify this, we obtained Pearson’s r of each graph, which is commonly used for linearity analysis, and which indicates higher linearity when it is closer to 1. All the graphs had very high Pearson’s r values, 0.997 or higher.

**Figure 8:**
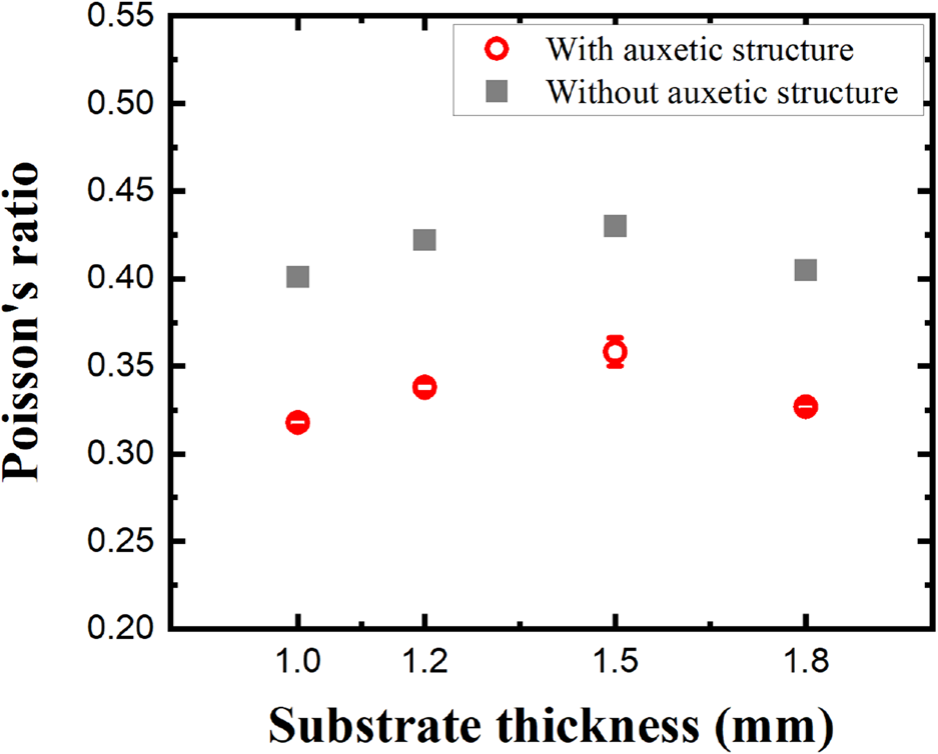
The variation of the Poisson’s ratio with the thickness of the planar substrate.

To determine the reduction rate of the Poisson’s ratio when the 500 μm thick re-entrant honeycomb structure was introduced and to verify the variation of biaxial strain-controlled characteristics with the thickness of the planar substrate, we compared the Poisson’s ratio for the thicknesses of the planar substrate as shown in Fig. 8. In literatures^44–46^, the Poisson’s ratio of planar PDMS has been estimated to be within the range of 0.4 to 0.5, and our results are consistent with this value, averaging around 0.415. With the introduction of the 500 μm thick re-entrant honeycomb structure, the Poisson’s ratios of the 1.0, 1.2, 1.5, and 1.8 mm thick PDMS substrates were reduced by 20.6%, 19.9%, 16.7%, and 19.3%, respectively; the reduction in the Poisson’s ratio was about 19.1% on average, and there was no significant difference by the reduction in the thickness of the planar PDMS substrate. If substrates with the 500 μm thick re-entrant honeycomb structure were to be used in solar cells, their area would be greater than the area of substrates without an auxetic structure by 9.44% upon being stretched. Thus, with the introduction of the 500 μm thick re-entrant honeycomb structure, the efficiency of substrates of stretchable solar cells of the same size was increased by 9.44%. Furthermore, the amount of materials required to fabricate substrates of stretchable solar cells with the same area after stretching to obtain the same efficiency was reduced by 8.60%. Therefore, if the suggested or improved re-entrant honeycomb structure are used in solar cells, their efficiency could be increased by more than 9.44% and material saving of more than 8.60% could be achieved.

## Conclusions

We presented a novel manufacturing technique for the production of biaxial strain-controlled substrates, which have significant potential for use in stretchable solar cells. The performance of these solar cells is directly related to their area, and thus the development of a reliable method for producing substrates with precise control over their biaxial strain is of utmost importance. To achieve this, we designed our stretchable substrates with an auxetic structure that exhibits a negative Poisson’s ratio. We then fabricated these substrates using a negative-patterned mold that was created through precise micro end-milling, followed by a replication process. The resulting substrates were found to have excellent control over their biaxial strain, making them ideal for use in stretchable solar cells. Our method is expected to have broad applications in the field of stretchable electronics, where precise control over the mechanical properties of materials is crucial.

## Data Availability

The datasets used and/or analysed during the current study available from the corresponding author on reasonable request.
